# Agents of change: establishing quality improvement collaboratives to improve adherence to Australian clinical guidelines for dementia care

**DOI:** 10.1186/s13012-018-0820-z

**Published:** 2018-09-24

**Authors:** Monica Cations, Maria Crotty, Janna Anneke Fitzgerald, Susan Kurrle, Ian D. Cameron, Craig Whitehead, Jane Thompson, Billingsley Kaambwa, Kate Hayes, Lenore de la Perrelle, Gorjana Radisic, Kate E. Laver

**Affiliations:** 1 College of Medicine and Public Health, Flinders University, Bedford Park, South Australia Australia; 2 Cognitive Decline Partnership Centre, The University of Sydney, Camperdown, New South Wales Australia; 3 Griffith Business School, Griffith University, Gold Coast, Queensland Australia; 4 Northern Clinical School, The University of Sydney, Camperdown, New South Wales Australia; 5 John Walsh Centre for Rehabilitation Research, Faculty of Medicine and Health, The University of Sydney, St Leonards, New South Wales Australia; 6 Healthcare and Hospital Process Improvement, Brisbane, Queensland Australia

**Keywords:** Quality improvement collaborative, Dementia, Guideline adherence, Implementation science, Aged care, Public involvement

## Abstract

**Background:**

Dissemination of clinical practice guidelines alone is insufficient to create meaningful change in clinical practice. Quality improvement collaborative models have potential to address the evidence-practice gap in dementia care because they capitalise on known knowledge translation enablers and incorporate optimal approaches to implementation. Non-pharmacological interventions focused on promoting independence are effective and favoured by people with dementia and their carers but are not routinely implemented. The objective of this translational project is to assess the impact of quality improvement collaboratives (QICs) on adherence to non-pharmacological recommendations from the Clinical Practice Guidelines for Dementia in Australia.

**Methods:**

This project will employ an interrupted time-series design with process evaluation to assess the impact, uptake, feasibility, accessibility, cost, and sustainability of the QICs over 18 months. Thirty clinicians from across Australia will be invited to join the QICs to build their capacity in leading innovation in dementia care. Clinicians will participate in a training program and be supported to develop and implement a quality improvement project unique to their service context using plan-do-study-act cycles. Regular online meetings with their peers in the QIC will facilitate benchmarking and problem-solving. Clinicians will describe their practice via monthly checklists, and guideline adherence will be determined against a set of defined criteria. Phone interviews with up to 180 client dyads will be used to assess satisfaction with care and client outcomes. Clinician interviews and field note data will be used to explore implementation and costs. Involvement of people with dementia and carers will be embedded in the study design, conduct, and reporting, in addition to clinical and industry expertise.

**Discussion:**

The quality of dementia care in Australia is largely dependent on the clinician involved and the extent to which they apply best available evidence in their practice. This study will determine the elements of this multifaceted implementation strategy that contributed to guideline adherence and client outcomes. The findings will inform future translational approaches to improving care and outcomes for people with dementia and their carers.

**Trial registration:**

Registered with the Australian New Zealand Clinical Trials Registry 21 February 2018 (ACTRN12618000268246).

## Background

Care for people with dementia and their carers is complex because of the condition’s multi-domain symptom profile, progressive and individualised course, and wide-reaching impact on the individual, their family, and the broader community [1]. In Australia and elsewhere, the quality of care received depends largely on the health professional involved and the extent to which they apply best available evidence in their practice [1–3].

The 2016 release of the *Clinical Practice Guidelines and Principles of Care for People with Dementia in Australia* (the Guidelines) included a systematic overview of evidence-based and best practice care that should be provided to people with dementia and informal carers (hereafter referred to as ‘carers’) in Australia [2]. However, dissemination of guidelines via promotion or word-of-mouth is insufficient to effect change in clinical practice [4]. Historically, implementation of clinical practice guidelines has occurred via two mechanisms: first, ‘early adopters’ attempt to implement recommendations in practice but do so in an unpredictable manner because they rarely have theoretical or methodological skills in implementation [5, 6]. Second, research teams conduct more rigorously designed implementation projects such as stepped wedge or cluster randomised trials in partnership with health or aged care services. Typically, these projects involve identification of barriers followed by the use of tailored interventions strategies which may include training, education, reminders, and audit and feedback [7]. Such projects usually focus on changing a single health professional behaviour and often result in only modest effects [8]. Additionally, sustainability of the change can be jeopardised when research resources are withdrawn [6].

More effective methods of guideline implementation are required based on the knowledge that health care professional behaviour is influenced by a wide range of personal and contextual factors. One systematic review identified 57 clusters of factors that played a role in professional practice [9]. In dementia care, barriers to knowledge translation include insufficient time to implement strategies; a lack of financial, leadership, or staff support; inadequate levels of knowledge or training; high staff turnover; inappropriate staffing or resources; lack of perceived ‘power’ in creating change; and previous unsuccessful attempts to implement change [3, 10]. Effective implementation of evidence in this context requires integrated, multimodal learning strategies that are tailored to the learner preferences, allow learners to ‘try-out’ new knowledge with expert follow-up, use simple messaging, provide incentives, and target the whole workplace rather than the individual health professional [3].

A quality improvement collaborative (QIC) is an innovative knowledge translation strategy incorporating these principles. Collaboratives bring together health professionals from multiple sites to facilitate learning about and sharing of methods to improve care. They generally include five elements: (1) focus on a specific healthcare topic, (2) participants from multiple sites, (3) a group of clinical and quality improvement experts available to guide the QIC members, (4) a set of structured activities to promote collaborative learning, and (5) a model for improvement that tracks progress against measurable aims [11, 12]. The QIC model is based on evidence that assessing one’s own progress and benchmarking with other professionals can facilitate faster and wider implementation of quality improvement practices [13]. QIC models have potential to address the evidence-practice gap in dementia care because they capitalise on known knowledge translation enablers: sufficient knowledge, access to feedback, a combined learning experience, formulating an incremental action plan, iterative practical experience with new knowledge, and realistic goal setting.

Quality improvement collaboratives have been successfully implemented to increase rates of breast feeding [14] and organ donation [15], reduce central line-associated bloodstream infection [16], and decrease post-stroke length of stay [17]. To our knowledge, QICs have not yet been used as an implementation strategy in community-based dementia care. Whether they are an accessible, feasible, cost-effective, and sustainable method of improving guideline adherence in dementia care is not known.

### Objectives

The primary aim of this project is to implement and sustain improvements in post-diagnosis care for people with dementia and their carers by increasing adherence to three key recommendations from the *Clinical Practice Guidelines for Dementia in Australia* [2]:

1. People with dementia living in the community should be offered occupational therapy (reflecting evidence-based programs)
2. People with dementia should be strongly encouraged to exercise
3. Carers and family of people with dementia should have access to programs that provide respite and support to optimise their ability to provide care for the person with dementia.

This will be achieved by establishing three nationwide QICs (of approximately ten health professionals and their sites each) who regularly work with people with dementia and their carers. The three guideline recommendations were chosen to be implemented because adherence to them is known to be poor. Occupational therapy intervention involving home modification, education, problem solving, and activity engagement is shown to be cost-effective [18], yet in practice occupational therapists focus on assessment at the expense of intervention [19]. People with dementia are not routinely encouraged to exercise or participate in physical activity [20] despite exercise being the most effective intervention demonstrated to delay functional decline [21]. Supporting carers of people with dementia to maintain their wellbeing and to independently problem solve and manage their own needs can reduce negative carer impacts as well as delaying functional decline and reducing the occurrence of changed behaviours in the person they care for [22, 23]. Yet these types of programs are not widely available, and carers report that they need more education, skills counselling, respite, and emotional support to help them in their caring role [24, 25]. Implementation of these guideline recommendations reflects the priorities of people with dementia and carers, who have called for improved post-diagnostic care which facilitates independence and social engagement for people with dementia and provides effective support for their carers [26]. The recommendations are low-cost, acceptable, and feasible interventions that reflect broader policies around healthy ageing [27].

The secondary aim of this project is to assess the impact of the QIC on experiences and outcomes for people with dementia and their carers.

The research questions are:

1. Can the establishment of a national dementia QIC increase adherence to three non-pharmacological recommendations from the guidelines? If so, are increases sustained?
2. How feasible is the establishment of the QICs?
3. What is the impact of the QIC on experiences and outcomes for people with dementia and carers?
4. What is the return on investment (cost-benefit) of establishing QICs?
5. How does participation in the QIC build knowledge and skills in quality improvement among the implementation clinicians?
6. How acceptable is the addition of quality improvement implementation skills and knowledge to clinicians’ existing skill sets, workload, and responsibilities?
7. What is the impact of involvement of people with dementia and carers in project design, conduct, and reporting?

## Methods

### Design

An overview of the project per guidelines by Proctor et al. [28] appears in Table 1. The impact of QICs on guideline implementation and outcomes for people with dementia and carers (‘client dyads’) will be evaluated in this implementation research project using an interrupted time-series design [29]. Interrupted time series is a strong evaluative design for estimating the impact of an intervention in non-randomised settings because it allows for detailed assessment of longitudinal trends associated with an intervention [30]. Feasibility, acceptability, cost-effectiveness, and sustainability of the model will be evaluated using an inbuilt mixed-methods process evaluation [31]. Both administrative data and data collected from participating health professionals, their employing organisations, and their clients with dementia and carers will inform the outcomes for this study.

**Table 1:** Overview of project per guidelines by Proctor et al. [28]

Action	Description
Name it	Establishment of QICs to improve care for people with dementia and their carers
Define it	QICs enable rapid, sustainable improvements in care by bringing together health services to learn together, share ideas, and benchmark outcomes
Specify it	
a) The actor	The project team establishes and supports the QICs; Implementation clinicians form the QICs and conduct quality improvement (using PDSA cycles)
b) The action	Completion of an online training course, development of a site-specific implementation plan, and enactment of this plan (using PDSA cycles)
c) Action target	“Implementation clinicians”: health professionals across Australia who have some leadership responsibilities yet are still closely connected to the delivery of services and can introduce changes to practice
d) Temporality	The clinicians will participate in online training, develop a sites-specific implementation plan, and then enact the plan.
e) Dose	Seven education modules of 2 hours each, to be completed over 8 weeks; 11 virtual QIC meetings
f) Implementation outcome affected	Primary outcome: adherence to recommendation as described in the criteria in Table 3.
g) Justification	The intervention was designed to match with factors known to enable evidence-based care for people with dementia and their carers and to be relatively ‘light touch’ and promote rapid change

*Abbreviations*: *PDSA* plan-study-do-act, *QIC* quality improvement collaborative

Participating clinicians will be taught and supported to undertake a quality improvement project using a framework modelled on the Institute for Healthcare Improvement Model for Improvement [32]. They will learn about key change management models, conduct stakeholder analysis, and assess their organisation’s readiness for change. They will use plan-do-study-act (PDSA) methods to make iterative and self-directed quality improvements. Iterative quality improvement methods allow for clinicians to learn by testing practice changes, rapidly assess their impact, and adapting according to feedback and reflection [33, 34].

### Context and setting

Formal support services for people with dementia in Australia are primarily delivered via hospitals and the Commonwealth subsidised aged care system [35]. Diagnosis occurs in primary care, specialist physician rooms, or hospital outpatient settings, but psychoeducation and service navigation and provision after this time vary. Therapy services to optimise function and independence are available in some, but not all, settings. People with dementia and their carers can access helplines and advisory services, and subsidised ongoing home care packages are available (based on need) with the primary aim of maintaining independence and delaying institutionalisation. Programs that provide respite for carers are available as well as short- or long-term care accommodation options, and these may include some access to regular physiotherapy, occupational therapy, and other allied health services. Younger people with dementia (under the age of 65) are eligible for disability support packages and can choose to move to the aged care system when they turn 65 or remain in the disability sector. Overall, service provision is fragmented and varies according to demographic, organisational, and policy factors [36–39]. We aim to recruit from a broad range of geographical and professional settings to gather a variety of perspectives about the acceptability and effectiveness of the QIC methodology.

### Participants

Participants in this project include the health professionals (*implementation clinicians*), their workplaces (*implementation sites*), and the people with dementia and/or carers to whom they provide service (*client dyads*).

Implementation clinicians will be health professionals across Australia who regularly work with people with dementia and/or their carers, have influence within their workplace (and possibly leadership responsibilities), and maintain a clinical workload. Implementation clinicians are sought from a variety of service contexts, professional backgrounds, and geographical locations. Recruitment will occur via targeted advertising with professional associations, aged care organisations, peak bodies, and health services. Clinicians who apply to join the QIC will be assessed for suitability based on their experience, seniority within their organisation, and existing caseload of people with dementia and/or carers. They will be required to demonstrate that they have the support of their management to participate. Eligible implementation clinicians will:

- Be medical, allied health, or nursing professionals registered with a professional body
- Regularly treat/work with people with dementia and/or their carers (i.e. at least twice a week)
- Have some influence within their workplace (e.g. via leadership responsibilities)
- Maintain a clinical workload of at least 30% of their working hours
- Give informed written consent
- Have signed approval to participate from their manager/supervisor

Implementation clinician workplaces will participate in the collaborative as ‘implementation sites’. An implementation site may include a general practice, a community care organisation, a day therapy centre, a memory clinic, a residential care facility, a hospital department, or any other organisation providing care for people with dementia. Direct managers/supervisors of implementation clinicians will participate in the process evaluation to gather their perspectives on the QIC and change management.

Client dyads will be existing patients with dementia and/or their carers within the caseload of the implementation clinician at the implementation site. Strict inclusion criteria will not be applied, and implementation clinicians will be asked to use their judgement; eligible people with dementia will be any client with a diagnosis of dementia (or suspected dementia) who attends a consultation with or without a carer. Carers will be any person attending the consultation who provides substantive care for a person with dementia and identifies as a carer. Implementation clinicians will complete the checklists about their consultation with each person with dementia (or carer, or dyad, where applicable) they see and return these to the study team. They will also ask for verbal assent (from both members of the dyad, where applicable) to pass contact details onto the study team for the purposes of two follow-up phone interviews. The study team will randomly select one dyad (per clinician) from among those assenting each month to receive these phone interviews (details below), for a total of 180 dyads.

### Intervention

The implementation strategy for this project was developed based on guidelines by Proctor et al. [28] and informed by the Johns Hopkins Quality and Safety Research Group translating evidence into practice model [40] (Table 2). It involves a comprehensive process of identifying candidates for QIC membership, planning and establishing the QICs, delivery of an evidence-based education package, provision of ongoing clinical and quality improvement expertise, regular financial and other incentives, and facilitation of QIC engagement over 18 months. The QIC model centres the health professional as the experts in their own service context and grants autonomy in enacting and tracking quality improvement activities over time.

**Table 2:** Overview of implementation strategy for Agents of Change project, informed by Straus et al. [40]

Implementation strategies	Description
Plan	
Gather information	• Literature review to establish known barriers and facilitators to implementation of evidence-based dementia care • Implementation clinicians conduct local needs assessment and organisational mapping • In-depth interviews with implementation clinicians and management • Establish steering committee with representation from people with dementia and carers to guide project conduct
Select strategies	• Implementation clinicians develop a formal implementation plan • Implementation clinicians develop tailored strategies to overcome barriers
Build buy-in	• Identify and prepare implementation clinicians • Involve organisation managers who confirm the clinicians’ involvement in the project and commitment to support • Involve members of the public (people with dementia and carers) and industry in all phases of the project
Initiate leadership	• Implementation clinicians identified as ‘Agents of Change’ within their organisation • Implementation clinicians establish ‘practice teams’ within their organisation to whom they will regularly report back and gather feedback
Develop relationships	• Build the QICs • Obtain formal research agreements • Develop partnerships between the implementation clinicians, members of the public (people with dementia and carers), industry, expert clinicians, and research team • One face-to-face start-up meeting
Educate	
Develop materials	• Development of MOOC with clinical content and focus on quality improvement in clinical settings • MOOC developed in consultation with people with dementia and carers, industry experts, educational designer • Development of implementation plan pro forma for clinicians • Establish group norms and standards of collaboration • Support for implementation clinicians to develop further site-specific resources
Educate	• Provision of training through seven-module MOOC • Phone orientation meeting with research team and face-to-face start up meeting to begin implementation plan brainstorming • Implementation plan reviewed by a person with dementia and their carer, quality improvement expert, and clinical expert • Support for implementation clinicians to gather feedback from ‘practice teams’ within their organisation • Regular audit and feedback based on clinician self-report and client dyad-report
Educate through peers	• Implementation plan reviewed by QIC peer • Ongoing communications within the QIC via online forums and monthly videoconferencing
Inform and influence stakeholders	• Use mass media, professional organisation newsletters, and industry publications to share information about the project and highlight implementation clinician plans
Restructure	• Implementation clinicians take a lead in quality improvement in their organisations • Site-specific implementation plan may involve restructuring or changes in structure, equipment, or records
Quality management	• Iterative quality improvement process using PDSA cycles • Ongoing peer supervision with subgroup of QIC members • Support for implementation clinicians to gather ongoing feedback from ‘practice teams’ within their organisation • Fidelity checking based on content of clinical interactions (via clinician self-report and patient and client dyad-report) • Monthly QIC meetings in which each clinician will report their plan activity for the month • Revisiting of implementation plan after each monthly meeting with update log; revised plan submitted 6 months after implementation • Reminders • Provision of client tools to increase uptake of best practice (to half of the sites) • Ongoing access to people with dementia and carers and clinical, quality improvement experts throughout implementation and follow-up
Finance	
Incentive scheme	• Implementation clinicians who complete 18 months follow-up receive access up to $1000 stipend to present their work at a meeting or conference • Provision of regular incentives to encourage fidelity (e.g. webinars and other resources, branded materials)

*Abbreviations*: *MOOC* massive open online course, *QIC* quality improvement collaborative

#### Plan

We will build the QIC by developing relationships with implementation clinicians and the managers of their organisations. Organisational support for the implementation clinician will be confirmed with a formal research agreement that will outline the expectations and role of the clinician, the site, and the research team. Detailed interviews with implementation clinicians and management, as well as organisational mapping and local needs assessment, will establish barriers to best practice care, opportunities for improvement, readiness for change, and expectations from the QIC. Implementation clinicians will be identified as change champions within their organisation via internal media and will be encouraged to establish a small team of colleagues with whom they can regularly report back on their project activity and gain feedback. This phase will also include development of partnerships with clinical, quality improvement and industry experts to provide guidance and advice throughout the life of the project. A once-off face-to-face meeting with all implementation clinicians, the research team, and clinical leaders will build buy-in, further develop relationships, and give credibility to the project.

People with dementia and/or carers will be recruited to be involved at all levels of the project including in the senior investigator and management teams, as members of an advisory committee, in intervention development workgroups, and for ongoing implementation clinician support. This involvement is embedded into the implementation strategy and wider project management across the life of the project to avoid tokenism [41] and to capitalise on demonstrated benefits for researchers, ethical and scientific standards, and the wider community [42–44]. Saunders et al. [45] argue that health research is a social process and should therefore be informed by interactions between researchers, research participants, and potential end beneficiaries (especially where the research will directly inform health care, as in this project). Feedback and ongoing support from people with dementia and/or carers are anticipated to contribute to project buy-in, motivation for change, and quality of plans for change among implementation clinicians [43, 46]. Recruitment for this purpose will be conducted separately from implementation sites via peak body and research centre networks. Per Australian guidelines [42], all of those recruited will be reimbursed for the time they spend providing expert advice and oversight.

#### Educate

Education for implementation clinicians will be delivered after a 9-month pre-intervention period and include written resources, webinars, expert feedback, collaboration and peer supervision, and online learning. The main component of the education will be an intensive, eight-module ‘massive open online course’ (MOOC) to upskill implementation clinicians on the clinical evidence base related to occupational therapy for people with dementia, physical activity for people with dementia, or carer support. The MOOC will focus on quality improvement techniques in clinical settings. Implementation clinicians will be guided through the development of an associated implementation plan unique to their service context and informed by service gaps and barriers and facilitators to improvement identified during the planning phase. The MOOC will be co-designed with people with dementia and carers to ensure it reflects their needs and experiences. Input will also be sought from clinical, aged care industry, quality improvement, and educational design experts to ensure it is rigorous, up-to-date, and effectively facilitates learning. Implementation clinicians will have access to people with dementia, carers, and clinical and quality improvement experts to review their plan and provide feedback. A peer review process will also allow implementation clinicians to give and receive feedback from another member of their QIC.

#### Restructure

Through their work to develop and implement a quality improvement activity to be delivered in their service, it is anticipated that the implementation clinician will become recognised as a clinical leader in their organisation. Their plan may include some restructuring of organisation policies, service delivery, resources, records, or staffing.

#### Quality management

Once reviewed, clinicians will implement their plan and participate in monthly virtual meetings with their QIC to benchmark and brainstorm strategies to overcome any noted roadblocks. They will iteratively review and update their plans using PDSA cycles [47] with support from people with dementia and carers and clinical and quality improvement experts. Clinician reports of the consultation will be audited, cross-referenced with client dyad reports, and anonymously fed back to facilitate self-assessment.

#### Finance

Travel costs for clinicians to attend the face-to-face meeting will be covered by the project. Regular incentives will be provided to encourage clinicians to remain engaged with the project and their implementation plan, including staggered provision of written resources (e.g. books, peer-reviewed journal articles), branded materials, gift cards, and exclusive webinars. The work by implementation clinicians to make clinical improvements will be highlighted by the research team in collaboration with their organisation in both mass media and internal organisation media. At the completion of their 18-month project commitment, implementation clinicians will have access to a $1000 stipend to attend a meeting or conference of their choice to present their work.

### Outcomes

The outcomes and measures that will be used for this study are presented in Table 3. Outcomes of interest relate to guideline adherence, implementation of the QIC methodology, service level effectiveness and harms, and client dyad outcomes.

**Table 3:** Project outcomes

Outcome domain	Details of measurement
Guideline adherence	
Exercise guideline adherence	Full adherence when: a) Clinician checklist explicitly references a discussion about current physical activity levels, and; b) Specific needs and barriers to physical activity are identified, and; c) Treatments/strategies recommended are clinical indicated based on needs/barriers, and; d) A written treatment plan for physical activity or exercise is provided to the person with dementia
Occupational therapy guideline adherence	Full adherence when: a) Home environment assessment has occurred (where applicable), and; b) Clinician checklist explicitly references identification of primary concern/s of person with dementia and carer, and; c) A written treatment plan to address needs of person with dementia and carer or give specific advice about suitable activities (that are tailored, of interest, and match capabilities) is provided
Carer support guideline adherence	Full adherence when: a) Clinician checklist explicitly references that the needs of the carer have been discussed during the consultation, and; b) Clinician checklist explicitly references clinically indicated provision of information about programs providing respite for the carer and/or other carer support services, and; c) A written treatment plan detailing key carer concerns and strategies to manage these is provided
Implementation	
Uptake	• Exposure: the extent to which clinicians use the materials and online training course • Initial use: initial changes in adherence to guideline recommendations
Sustainability	• Continued changes in adherence to guideline recommendation.
Feasibility	• Recruitment: attraction of implementation clinicians and participating organisations • Consent rate for people with dementia and their carers agreeing to be contacted for follow-up • Maintenance: involvement in the program and contribution to data collection • Withdrawals
Acceptability	• Interviews with implementation clinicians regarding participation in the program and the acceptability of the intervention and process • QIKAT-R tool: a three-item tool that identifies the skills and knowledge of the implementation clinicians in quality improvement (i.e. how well they can assess the need for change and identify appropriate strategies) • NOMAD tool: a validated method of exploring why clinicians change their practice and why they do not, and this is a key aim of process evaluation.
Fidelity	• Fidelity determined via checklists on the content of clinician-patient/carers interactions. Data captured via clinician self-report checklist and phone call surveys with patients and carers
Penetration	• Context: information about the sites and funding models, as well as the different types of clinicians (professional background, level of seniority, and type of role). • Reach: does the project reach a variety of different sites and people with dementia and carers
Costs	• Calculation of costs of providing the intervention (personnel, technology, stipends, development and distribution of educational materials) and in-kind contribution required for each site estimated using a ‘bottom-up’ micro-costing approach. • Willingness to pay questionnaire
Impact of involvement of people with dementia and carers at all levels of the project	• Impact of involvement of people with dementia and carers on intervention quality, success • Expectations and experiences of people with dementia and carers involved in the project
Service	
Safety	• Implementation clinicians will record any adverse events and discuss any unintended consequences
Client	
Satisfaction	• Amended version of the Patient Satisfaction Questionnaire Short-Form
Function/QOL	• DEMQOL assesses the quality of life of clients with dementia (exercise and OT groups only) • ZBI assesses the burden experienced by carers of people with dementia (‘carer support’ group only)

*Abbreviations*: *DEMQOL* Dementia Quality of Life Questionnaire, *MOOC* massive open online course, *NOMAD* questionnaire tool based on Normalisation Process Theory, *OT* occupational therapy, *QIKAT-R* Quality Improvement Knowledge Application Tool Revised, *QOL* quality of life, *ZBI* Zarit Burden Interview

#### Guideline adherence

The primary outcome of the implementation evaluation is changes in guideline adherence over time. Guideline adherence will be assessed using monthly checklists completed by implementation clinicians about their consultations with people with dementia and/or carers. Clinicians will complete the checklists for the first ten consecutive consultations each month. Clinicians will be asked to provide a ‘snapshot’ of the consultation including its purpose, content, and outcomes. A process for guideline adherence scoring was modelled on methods used in Kortekaas et al. [48] and van Fenema et al. [49]. Key indicators of guideline adherence were developed in consultation with clinical and consumer experts (see Table 3). Two independent researchers will rate whether the practice reported by the clinician was inadequately (− 1), partially (or unclear; 0), or fully adherent (+ 1) to the relevant recommendation. In cases of disagreement, a third external clinical academic will be contracted to make a final decision. A follow-up phone interview with a random selection of client dyads each month up to 5 weeks after the consultation will be used to verify these reports, and ‘agreement’ between the client dyad and clinician will be assessed. Phone interviews will be conducted by the study team with both the person with dementia and their carer where possible, or just the carer where they are directly participating as a client of clinicians in the ‘carer support’ or the person with dementia is unable to participate in a phone call. People with dementia who attend the consultation alone (including those who live in long-term care) will not be contacted by phone. Interviews will gather perspectives from the dyad or carer about their recollection of the consultation and the extent of guideline adherence from their perspective. Clinician and client dyad data will be triangulated with field notes from QIC meetings, online message board participation, and other contact with the research team.

#### Process evaluation

Feasibility and acceptability among service providers of the QIC model for improving service provision for people with dementia and carers will be assessed by tracking the level of interest from potential implementation clinicians and following up with those who originally expressed interest but declined participation after receiving further information to identify key barriers. We will also track the consent rate of client dyads agreeing to be contacted by phone following the consultation to determine acceptability of this method of data collection. In-depth interviews with implementation clinicians and their managers early in the project will establish expectations, perceived acceptability of the QIC, potential barriers to participation, current practice, organisational cultures, and previous experiences with innovation. Interviews will be repeated at the end of the 18 months to understand their experience of the QIC and the education package and factors that influenced their uptake. The interview questions were developed based on the Consolidated Framework for Implementation Research qualitative interview guide, developed to capture the many constructs known to be important to implementation success [50].

Interview data will be supplemented with the 23-item NoMAD survey instrument based on Normalisation Process Theory, completed by implementation clinicians to assess their perception of the integration of their quality improvement plan [51]. Practical knowledge of the implementation clinicians in quality improvement will be measured using vignettes and the Quality Improvement Knowledge Application Tool Revised [52]. Detailed field notes related to project acceptability, feasibility, and sustainability will be kept and analysed including email, online messaging, phone, and face-to-face contact between the implementation clinicians and research team.

Costs associated with establishing and running the QIC will be estimated. Total costs include costs of providing the intervention (personnel, technology, stipends, and development and distribution of educational materials) and in-kind contribution required for each site estimated using a ‘bottom-up’ micro-costing approach. Costs will be estimated using administrative data and resource use questionnaires administered to key implementation site personnel. The monetary benefits of adopting and implementing the QIC, from implementation clinicians’ point of view, will be determined using contingent valuation techniques [53]. This technique allows for a monetary value to be placed on a good or service that is not yet available in the marketplace. The maximum amount of money that implementation clinicians would be willing to pay for the perceived benefits (buying price) of implementing the QIC will be estimated using their responses to a willingness to pay (WTP) questionnaire. As per best practice guidelines [53, 54], the WTP questionnaire will (a) identify the benefits that are likely to be realised from the QIC, (b) assess prior knowledge about QIC and attitudes toward it, and (c) establish respondents’ WTP.

Finally, implementation clinicians and managers will complete an organisational network map of their implementation site to describe the structure of their services, relationships between staff members, and potential sources and supporters of innovation. These maps can be used to examine the complex interactions between structures and people that might not be captured in an interview [55]. Maps will also be used to examine the penetration of the project in terms of the variety of sites, funding models, professional backgrounds, level of seniority, and types of roles engaged with the QICs. Clinician checklists, interviews, client dyad phone calls, and field notes will be examined to assess whether participation improved the reach of services to previously underserviced clients.

#### Involvement of people with dementia and carers

We will assess the impact of involvement of people with dementia and carers in the study design, conduct, and reporting on the quality of the intervention (from the perspective of clinicians) during the process evaluation. The impact of involvement in research of people directly affected by the conditions being researched on research quality and outcomes is underreported [56], and knowledge of impact is important to establishing best practice and policy directives [45, 56]. Modelled on Dudley et al. [46], qualitative interview questions will be included to assess implementation clinicians’ perspectives on the value of the contributions of people with dementia and carers, impact on clinicians’ learning and quality improvement activities, and any negative impacts. Results will be reported per recommendations by Staniszewska et al. [57].

#### Service and client-level outcomes

During the client dyad phone interview up to 5 weeks after the consultation, both the person with dementia (where applicable) and their carer will be asked to rate their satisfaction with the consultation using an amended version of the Patient Satisfaction Questionnaire Short-Form (PSQ-18) [58]. Seven PSQ-18 items were selected because they were relevant to the types of consultations delivered by QIC clinicians. They assess satisfaction with the time spent with the health professional and their communication and interpersonal manner on a 5-point Likert scale (total score range 7–35). Items on the PSQ-18 have adequate internal consistency (all >  0.65) [58].

Adverse events that may reflect the safety of the QIC model will be reported by implementation clinicians during monthly QIC meetings and in in-depth interviews. Client dyads will also be asked to reflect on the recommendations made during the consultation by the implementation clinician and report any negative consequences.

We will also assess the impact of the QIC model and guideline adherence on quality of life for the person with dementia (clients of clinicians in the exercise and occupational therapy QICs) or burden for the carer (clients of clinicians in the ‘carer support’ QIC) during this phone interview. These outcomes will be assessed a second time with a follow-up phone interview up to 7 weeks after the first, to identify sustained impact.

Quality of life will be assessed using the DEMQOL-Proxy [59], a 31-item questionnaire administered with the carer. The DEMQOL-Proxy asks the carer to report the extent to which the person with dementia has exhibited a variety of emotions and functional behaviours in the past week on a 4-point Likert scale, as well as a global quality of life item. Scores are summed to a total of 31–124, with higher scores indicating better QOL. The DEMQOL-Proxy has demonstrated good discriminant validity and converges well with the non-dementia-specific EQ-5D-5L [60]. The shortened 12-item version of the Zarit Burden Interview will be used to establish and monitor carer burden for client dyads of clinicians in the ‘carer support’ QIC. This shortened version correlates well with the original 21- and 22-item versions [61] and has high internal consistency (*α* = 0.87) and discriminant validity (AUC = 0.99) [62]. Carers are asked to report the frequency of their feelings of stress and burden associated with caring for the person with dementia on a 5-point Likert scale, summed to a maximum score of 48.

### Analysis

#### Quantitative analysis

Guideline adherence and client outcomes over time will be evaluated with a segmented regression analysis using the PROC NLIN function of SAS version 13.2 [63]. This technique uses modelling to draw conclusions about an outcome (in this case guideline adherence) across distinct segments of time (in this case, before and after quality improvement implementation) [64]. Data points for the time series will be the extent of guideline adherence each month over 18 months. Potential confounding variables will be fitted as covariates, and the most parsimonious model will be determined via stepwise backward elimination. The hypothesised outcomes of interest for this study are level and trend changes reflecting increasing adherence to the relevant guideline recommendation after the intervention (post-education quality improvement implementation) and over the 18 months. We will also calculate the counterfactual value and its proportionate distance from the actual estimated value [65]. The PROC AUTOREG function will be used to control for autocorrelation [63].

The sample size calculation for segmented regression analysis is related to the estimated number of time points at which data will be recorded. It is necessary to have enough time points before and after the intervention. This study incorporates 18 months of data collection (9 months pre-intervention and 9 months post-intervention) and is powered at 83% to detect a minimum 15% change in guideline adherence based on an estimated effect size of 1, autocorrelation of 0.3, and *α* = 0.05 [66]. This change in guideline adherence was used for power analysis based on literature suggesting an average of 10–15% improvement in adherence from traditional guideline dissemination activities [7]. Per recommendations from Wagner et al. [65], clinicians will submit up to ten checklists for each data point in the time series (for a total of up to 300 checklists each month) to achieve an acceptable level of variability of the estimate at each time point. The study design therefore meets the criteria for a robust interrupted time-series [67].

Feasibility data elicited from field notes and records will be provided descriptively so that it is possible to determine how many people expressed interest, how many formally participated, and how many people withdrew (and reasons for withdrawal). We will present information about the characteristics of the clinicians and their workplaces. We will also describe engagement and exposure to the intervention through presentation of time spent participating in the online training and participation in other components of the intervention such as number of contributions to the online community of practice and completion of the implementation plan. Data from the NOMAD and QIKAT tools regarding implementation readiness and proficiency will be presented descriptively (with means and standard deviations where appropriate).

The mixed sources of data will be used to explore factors underlying successful implementation. The percentage increase in average guideline adherence during the 9-month pre- and post-intervention periods will be calculated for individual clinicians, to represent implementation success. *T* or correlation tests (where appropriate) will be used to identify the impact of workplace characteristics, time engaged in the intervention, and NOMAD/QIKAT scores on implementation success. Qualitative data will be used to explore and contextualise the findings.

A cost-benefit analysis will be used for the economic evaluation [54]. The costs associated with establishing and running the QIC will be compared to the monetary benefits of implementing this strategy. The QIC will be considered value for money (i.e. cost-beneficial) if benefits exceed costs. Benefits will be considered from the perspective of implementation clinicians’ point of view. The return on investment will also be estimated as the ratio of benefits divided by total costs of the intervention (i.e. the benefit-cost ratio) [54].

#### Qualitative analysis

Qualitative interview and field note data will be transcribed verbatim and entered into QSR NVivo version 10 [68], and two people will code the data. A combination of inductive and deductive thematic analysis will be used to identify themes within the data related to implementation of quality improvement programs, organisational culture and innovation, evaluation of the QIC model, and key barriers and facilitators to guideline adherence [69] The structure of the interview (based around questions from the Consolidated Framework for Implementation Research guide) will assist with linking the findings with theoretical models though we will not restrict our themes to those described in the model.

## Discussion

This implementation research project seeks to examine the efficacy of establishing QICs to improve adherence to key evidence-based clinical guidelines for dementia care. To our knowledge, this is the first study to implement QICs to improve the quality of non-pharmacological care programs for people with dementia and carers living in the community. Dementia service provision is highly complex and is largely dependent on the knowledge, skills, and resources available to the health professional. Quality improvement collaboratives are an innovative method of implementation science that address known barriers to adherence to evidence-based clinical guidelines, including a lack of perceived skills in quality improvement and insufficient clinical support [3, 10].

This study benefits from several strengths. The intervention is low-cost and ‘light-touch’ in that it centres practising clinicians as experts in their own service and supports them to become leaders in effecting change. The mechanisms for embedding change are pragmatic and draw on theories of implementation and quality improvement methodology. The implementation sites and clinicians are diverse, and thus, the project is not susceptible to changes in the policy or funding environment. Time series designs are the strongest quasi-experimental designs for estimating effects of an intervention where randomisation is not possible. Segmented regression analysis of time series data can provide insights into the dynamics of change while controlling for prior trends in the outcome [65].

Despite these strengths, the approach for this study has some important limitations. First, the inclusion of a control group was considered unethical because clients would be deprived of best-practice care, and engaging clinicians to provide data without any intervention would be difficult. Effects occurring at the same time but separate to the intervention will not be separated and controlled for, threatening validity. Nonetheless, even without a control group, segmented regression analysis makes multiple assessments of the outcome and therefore addresses important threats to internal validity. Second, the primary outcome measure (guideline adherence) will be self-reported by the implementation clinicians and is therefore vulnerable to a responding bias. The triangulation of data from client dyad phone calls will help to address this problem, and adherence (according to the criteria described in Table 3) will be independently judged by two members of the research team and an external third party where needed based on clinician reported ‘snapshots’ of the consultation. Nonetheless, some responding bias may still exist. Third, a selection bias may be present in the participating implementation clinicians. The ‘opt-in’ approach to recruitment will likely lead to a group of passionate and engaged clinicians who may not represent the wider population of clinicians working with people with dementia and their carers. Finally, there are some limitations associated with segmented regression analysis. These models assume a linear trend in the outcome within each segment, but this may not hold over longer intervals [65]. Segmented regression analysis also does not allow for statistical controlling of individual-level covariates. However, these covariates will only become confounding where they both predict the outcome and change in relationship to the time of the intervention. No such covariates are anticipated.

Clinical guidelines aim to promote evidence-based practice, improve patient outcomes, and allow more efficient use of resources [70]. However, dissemination of guidelines alone is insufficient to effect change in clinical practice. This study will identify the elements of a multifaceted implementation strategy that contributed to improved guideline adherence, client outcomes, and clinician skills. Outcomes will inform large-scale strategies to promote professional and organisational innovation and effect sustainable improvements to the quality of dementia care more widely.

## Abbreviations

MOOC: Massive open online course
PDSA: Plan-do-study-act
PSQ-18: Patient Satisfaction Questionnaire Short-Form
QIC: Quality improvement collaborative
WTP: Willingness to pay
ZBI: Zarit Burden Interview
